# Contribution of macrophages in the contrast loss in iron oxide-based MRI cancer cell tracking studies

**DOI:** 10.18632/oncotarget.17103

**Published:** 2017-04-13

**Authors:** Pierre Danhier, Gladys Deumer, Nicolas Joudiou, Caroline Bouzin, Philippe Levêque, Vincent Haufroid, Bénédicte F. Jordan, Olivier Feron, Pierre Sonveaux, Bernard Gallez

**Affiliations:** ^1^ Louvain Drug Research Institute, Biomedical Magnetic Resonance Research Group, Université Catholique de Louvain (UCL), Brussels, Belgium; ^2^ Louvain Center for Toxicology and Applied Pharmacology, Université Catholique de Louvain (UCL), Brussels, Belgium; ^3^ Institut de Recherche Expérimentale et Clinique (IREC), IREC Imaging Platform, Université Catholique de Louvain (UCL), Brussels, Belgium; ^4^ Institut de Recherche Expérimentale et Clinique (IREC), Pole of Pharmacology, Université Catholique de Louvain (UCL), Brussels, Belgium

**Keywords:** MRI, EPR, cell tracking, cancer metastasis, iron oxides

## Abstract

Magnetic resonance imaging (MRI) cell tracking of cancer cells labeled with superparamagnetic iron oxides (SPIO) allows visualizing metastatic cells in preclinical models. However, previous works showed that the signal void induced by SPIO on T_2_(*)-weighted images decreased over time. Here, we aim at characterizing the fate of iron oxide nanoparticles used in cell tracking studies and the role of macrophages in SPIO metabolism.

*In vivo* MRI cell tracking of SPIO positive 4T1 breast cancer cells revealed a quick loss of T_2_* contrast after injection. We next took advantage of electron paramagnetic resonance (EPR) spectroscopy and inductively coupled plasma mass spectroscopy (ICP-MS) for characterizing the evolution of superparamagnetic and non-superparamagnetic iron pools in 4T1 breast cancer cells and J774 macrophages after SPIO labeling. These *in vitro* experiments and histology studies performed on 4T1 tumors highlighted the quick degradation of iron oxides by macrophages in SPIO-based cell tracking experiments.

In conclusion, the release of SPIO by dying cancer cells and the subsequent uptake of iron oxides by tumor macrophages are limiting factors in MRI cell tracking experiments that plead for the use of (MR) reporter-gene based imaging methods for the long-term tracking of metastatic cells.

## INTRODUCTION

Metastasis is the leading cause of cancer-related death [1]. Despite the development of new effective anticancer treatments, metastases are still difficult to treat. Hence, it is crucial to develop preclinical imaging tools for monitoring the metastatic cascade and for understanding its features *in vivo*.

Cell tracking or cellular imaging is defined as the “non-invasive and repetitive imaging of targeted cells and cellular processes in living organisms” [2]. Applications of cellular imaging include the monitoring of stem cells in cell-based therapies and the visualization of metastatic cancer cells in small animals [3]. In magnetic resonance imaging (MRI) cell tracking studies, cells are first labeled *in vitro* with contrast agents prior to their injection *in vivo*. Superparamagnetic particles of iron oxides (SPIO) are widely used in cell tracking studies because (*i*) they induce signal voids on T_2_- and T_2_*-weighted images (T_2_^(*)^-w), (*ii*) they are biocompatible and (*iii*) they are easily incorporated in the cell cytoplasm [2, 4, 5].

Previous studies showed that MRI allows single cell detection on high-resolution images [6–8]. For instance, Heyn et *al*. reported the imaging of isolated breast cancer cells disseminated in the mouse brain and the subsequent development of macrometastases [9]. Since the SPIO contrast is diluted with cell division, previous works performed on melanoma and breast tumor models suggested that the signal void persistence indicated the presence of quiescent cancer cells, whereas regions of contrast loss indicated proliferative tumor cells [10, 11]. For these reasons, MRI offers great promises for monitoring the development of metastases of clonal origin and monitoring dormant metastatic cells [9, 10, 12]. In the clinic, dormant cancer cells are nonresponsive to therapy and are responsible for recurrences [13]. These perspectives are however challenged from other observations. In cell-based therapy approaches, MRI cell tracking experiments failed to characterize the long-term fate of stem cells. Several immunohistochemistry studies performed several weeks after cell delivery showed that macrophages take up SPIO released by dying cells. Our works previously showed that the signal void induced by SPIO-labeled cancer cells on MRI scans quickly decreased at the injection site and at metastatic locations [8, 14]. Moreover, we showed that the iron oxide content in tissues decreased with time [8, 14]. We therefore hypothesized that *(i)* dilution of SPIO with cell division, *(ii)* SPIO metabolism by macrophages recruited to the tumor site and *(iii)* clearance of SPIO from dead cells could explain the loss of contrast and/or the drop of SPIO content in tissues [8, 14]. Hence, the evolution of SPIO contrast may be influenced by the proliferative status but also by the phagocytic activity of tumor macrophages.

Here, we aimed at characterizing the role of macrophages in SPIO uptake and degradation *in vivo*. It will allow determining if MRI cell tracking can be used for assessing the proliferative status of tumor cells *in vivo*. MRI was performed to characterize the fate of SPIO-labeled breast cancer cells *in vivo*. We next questioned the *in vitro* fate of iron oxides after intracellular incorporation in breast cancer cells and macrophages. We took advantage of the superparamagnetic (SP) properties of these nanoparticles, and used electron paramagnetic resonance (EPR) spectroscopy for measuring superparamagnetic iron. EPR was validated in previous studies for characterizing the SPIO content of cells and tissues [14–22]. Inductively coupled plasma mass spectroscopy (ICP-MS) served for the sensitive quantification of total iron pools (SP + non-SP) [23]. Correlating both ICP-MS and EPR results provided important information on the degradation of iron oxides after SPIO labeling in breast cancer cells and macrophages.

## RESULTS

**Figure 1:**
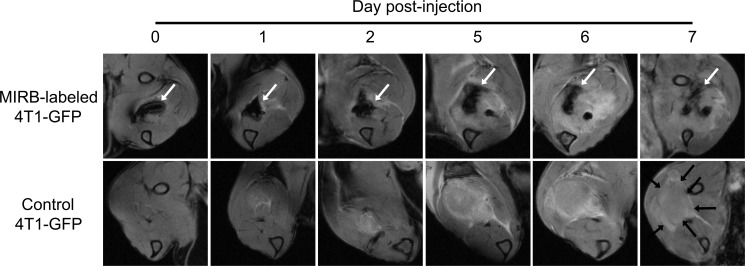
MRI shows that SPIO-induced signal voids progressively disappear after intramuscular injection of iron oxide-labeled 4T1 cells Mice received an intramuscular injection of 10^6^ MIRB-labeled 4T1-GFP cells (upper images) or 10^6^ SPIO free 4T1-GFP cells (lower images). Longitudinal T_2_*-w images of MIRB-labeled 4T1-GFP cells (upper images), where the negative contrast due to the presence of SPIO-labeled cells over time is indicated by white arrows. T_2_*-w images of control 4T1-GFP cells (lower images), highlighting the absence of signal voids due to SPIO and the apparition of a hyperintense area due to tumor growth (black arrows) (*n* = 4).

Using MRI (11.7 T), we first tracked green fluorescent protein-tagged 4T1 (4T1-GFP) cells labeled with Modlay Ion Rhodamine B (MIRB) SPIO *in vivo*. Figure 1 shows that an intramuscular injection of SPIO-labeled 4T1-GFP cells in mice induced a strong signal void on T_2_*-w images, whereas the injection of control 4T1-GFP cells was not detected on MR scans. In the following days, the hypointense spot progressively disappeared (Figure 1). We also noticed the appearance of a hyperintense area in both groups that was attributed to the development of the tumor (Figure 1).

**Figure 2:**
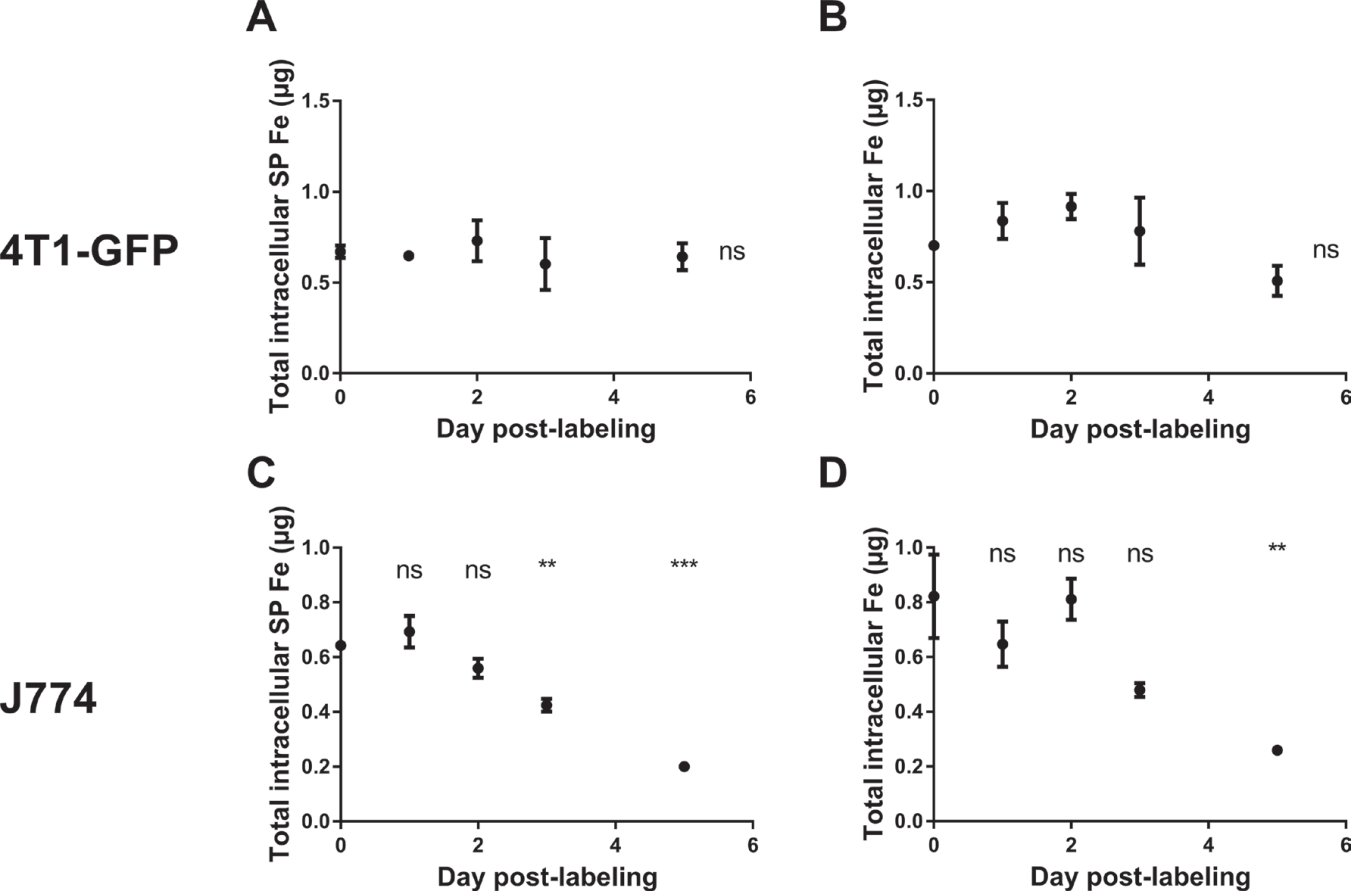
The superparamagnetic iron content remains constant in 4T1-GFP cells after MIRB labeling, whereas it drops in J774 macrophages (**A**) The SP iron pool measured by EPR and (**B**) the total iron (SP + non-SP) pool measured by ICP-MS were quantified in MIRB-labeled 4T1-GFP breast cancer cells. (**C**) The SP iron pool measured by EPR and (**D**) the total iron (SP + non-SP) pool measured by ICP-MS were quantified in MIRB-labeled J774 cells. Data are expressed as means ± SEM. **p < 0.01, ***p < 0.001, ns, *p* > 0.05.

We next aimed at characterizing the role of macrophages in the loss of contrast observed on MR scans. For this purpose, we next measured the evolution of SP iron content and total (SP + non-SP) iron content in 4T1-GFP cells and J774 macrophages after SPIO labeling. In the total population of MIRB-labeled 4T1-GFP breast cancer cells, SP iron levels were stable up to five days after labeling (Figure 2A, 0.67 ± 0.03 μg SP iron at day 0 *versus* 0.64 ± 0.07 μg SP iron at day 5, *p* = 0.9984). No difference in total iron levels (SP + non-SP) between groups was detected (Figure 2B, 0.70 ± 0.01 μg Fe at day 0 *versus* 0.51 ± 0.08 μg Fe at day 5, *p* = 0.53). Conversely, intracellular SP iron oxide content progressively decreased in J774 macrophages after MIRB labeling (Figure 2C, 0.64 ± 0.02 μg SP at day 0 *versus* 0.20 ± 0.01 μg SP iron at day 5, *p* < 0.001). Similarly, total (SP + non-SP) iron levels decreased in MIRB-labeled J774 cells after SPIO labeling (Figure 2D, 0.82 ± 0.15 μg iron at day 0 *versus* 0.26 ± 0.01 μg iron at day 5, *p* = 0.0031).

**Figure 3:**
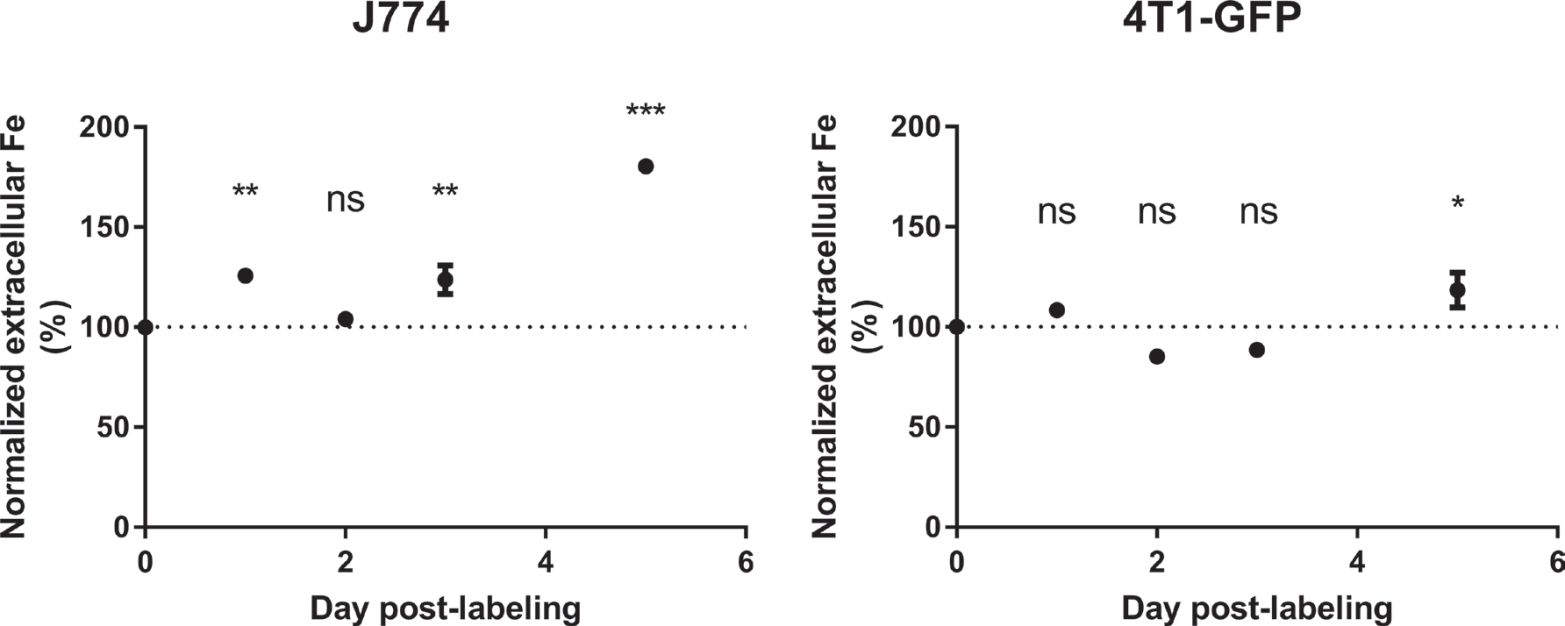
Extracellular iron levels increase in J774 macrophages after MIRB labeling The extracellular iron levels were measured by ICP-MS in both J774 and 4T1-GFP cells labeled with MIRB. Data are expressed as means ± SEM. *n* = 3. *p < 0.05, **p < 0.01, ***p < 0.001, ns, *p* > 0.05.

**Figure 4:**
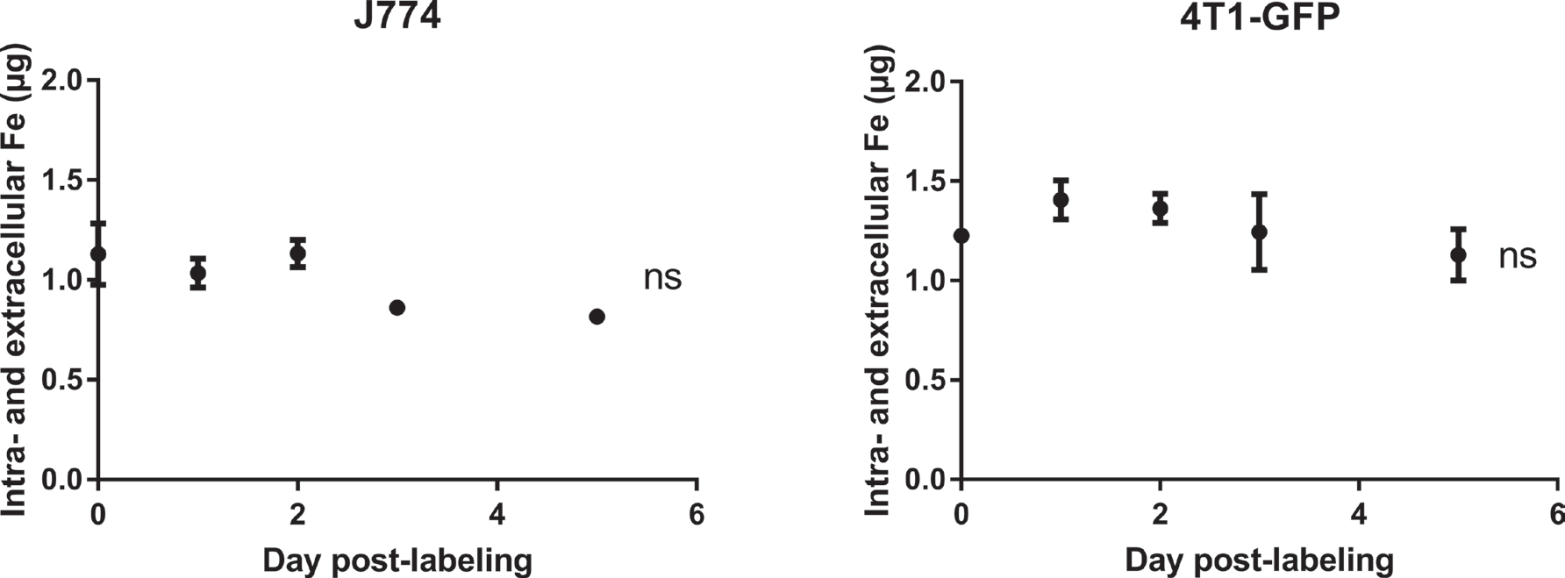
The intra- and extracellular iron pools do not vary with time after SPIO labeling in both 4T1-GFP and J774 cells Iron values, obtained by ICP-MS, of cell samples and extracellular media were pooled. Data are expressed as means ± SEM. *n* = 3, ns, *p* > 0.05.

These *in vitro* experiments showed that the intracellular (SP) iron content dropped in J774 macrophages but not in 4T1-GFP cells after MIRB labeling. It suggested that macrophages in particular metabolize SPIO. Using ICP-MS, we therefore compared iron release by J774 and 4T1-GFP cells after SPIO incubation. Figure 3 shows that J774 macrophages released significant amounts of iron in the culture medium after MIRB-labeling (Figure 3, 0.31 ± 0.01 μg iron in MIRB-labeled J774 cells at day 0 cells *versus* 0.56 ± 0.01 μg iron at day 5, *p* < 0,0001). Comparatively, extracellular iron concentration only slightly increased in the 4T1-GFP + MIRB group (Figure 3, 0.52 ± 0.01 μg iron in MIRB-labeled 4T1-GFP cells at day 0 cells *versus* 0.62 ± 0.05 μg iron at day 5, *p* = 0.036). Of note, we did not detect any EPR signal coming from extracellular media of cultured 4T1-GFP + MIRB or J774 + MIRB cells (data not shown). When both total intracellular and extracellular iron levels (SP + non-SP) are pooled, no significant variation in iron content was detected in 4T1-GFP and J774 macrophages (Figure 4).

**Figure 5:**
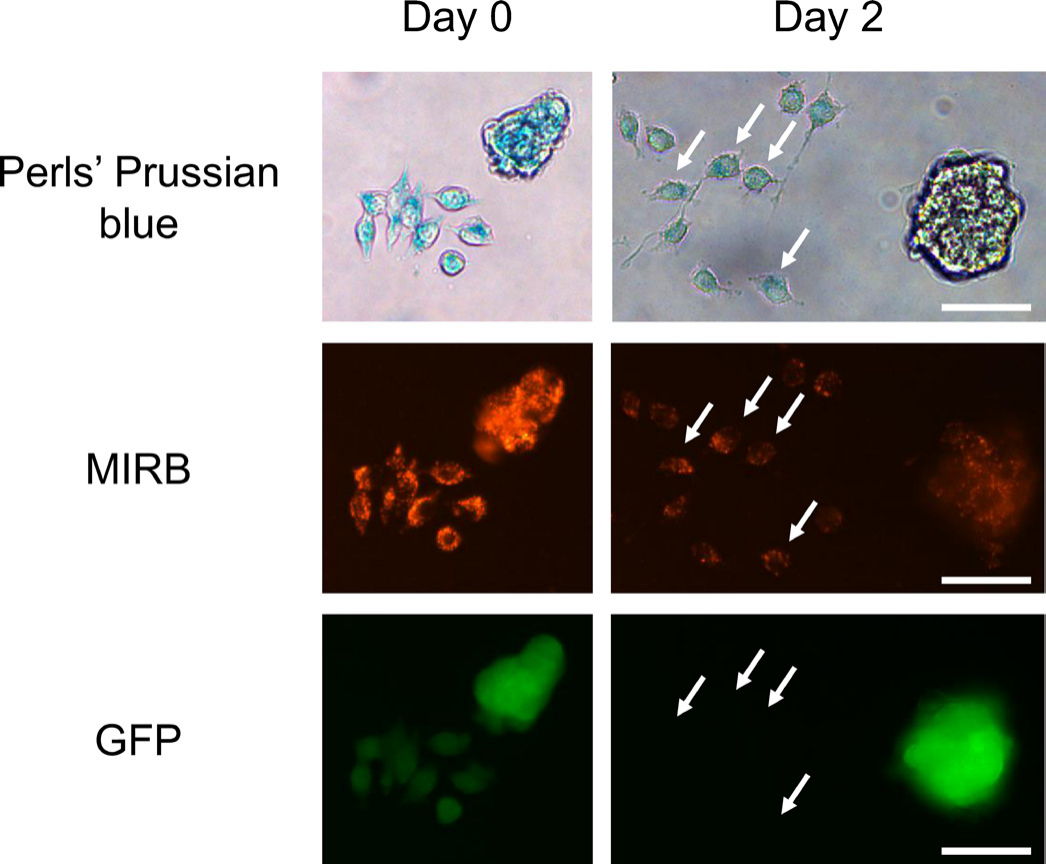
SPIO-free J774 macrophages take up iron oxides released by MIRB-labeled 4T1-GFP cells Fluorescence imaging of MIRB iron oxides (red fluorescence) and 4T1-GFP cells, combined with the iron detection using Perls'Prussian blue staining were performed just after overnight incubation of 4T1-GFP cells (day 0) with SPIO. GFP/SPIO negative J774 macrophages were added to SPIO-labeled 4T1-GFP cells and allowed to grow for 2 days before imaging (day 2). White arrows indicate the presence of GFP negative J774 macrophages containing red fluorescent SPIO. Scale bar, 50 μm.

**Figure 6:**
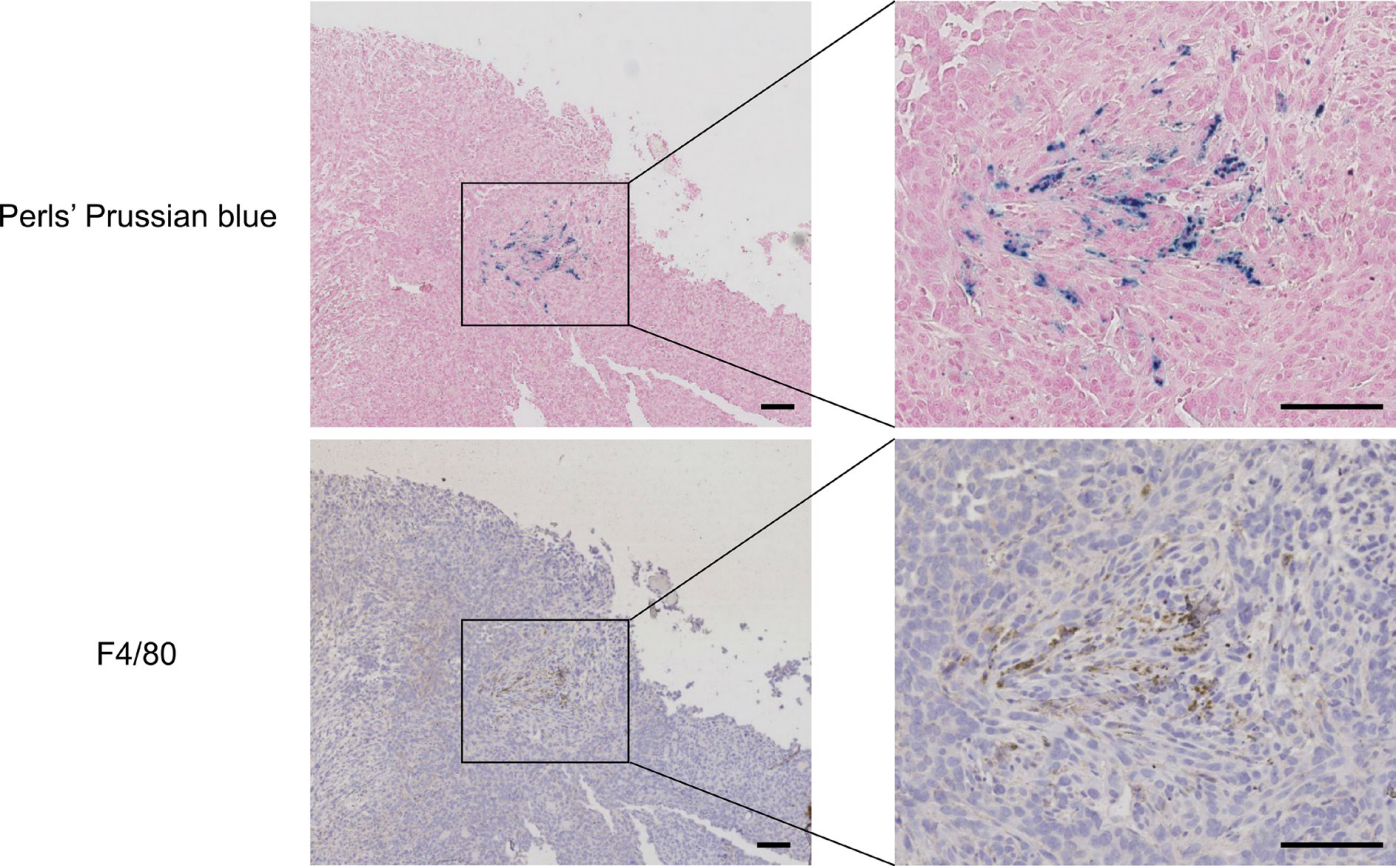
Histology of tumor tissue sample at day 7 post-injection of MIRB-labeled 4T1-GFP cancer cells shows that remaining SPIO are engulfed by macrophages Mice received an intramuscular injection of 10^6^ MIRB-labeled 4T1-GFP cells and underwent MRI scans for 7 days. After longitudinal MR imaging, mice were sacrificed and tumors were collected for histological analysis. Microscopy pictures show iron positive cells (Perls' Prussian blue staining) and F4/80-positive macrophages (brown). Scale bar, 100 μm.

We addressed the fate of intracellular SPIO when MIRB-labeled 4T1-GFP cells are grown in the presence of control J774 macrophages. For this purpose, SPIO negative/GFP negative J774 macrophages were added to SPIO positive 4T1-GFP cells (overnight incubation with MIRB nanoparticles). Microscopy pictures performed just after incubation with MIRB nanoparticles showed that 4T1-GFP cells incorporated red fluorescent SPIO (Figure 5). At day 2 after the addition of J774 macrophages to MIRB positive 4T1-GFP cells, we observed that the red fluorescence decreased compared to day 0 and co-localized with GFP negative J774 macrophages (Figure 5). Of note, Perls' Prussian blue staining confirmed that red fluorescence matched with iron clusters (Figure 5). Moreover, Perls' Prussian blue staining and immunohistochemistry studies performed on 4T1 tumors after MRI cell tracking (See Figure 1) at day +7 showed that remaining iron clusters colocalized with F4/80 positive macrophages (Figure 6).

## DISCUSSION

MRI cell tracking has several potential applications related to cancer. In a series of studies, it was used for characterizing the interplay between immune cells (dendritic cells, T cells and NK cells) and cancer cells for the evaluation of cancer immunotherapy treatments [24–26]. SPIO-based MRI cell tracking can also be used for monitoring the fate of cancer and tumor-associated cells. For instance, cellular imaging techniques were used for visualizing the homing of macrophages into metastasized lymph nodes and the trafficking of endothelial progenitor cells to sites of tumor angiogenesis [27, 28].

We previously used magnetic cell tracking techniques in experimental breast cancer brain metastasis and renal cancer models [8, 14]. These experiments evidenced that the negative MR T_2_(*) contrast induced by SPIO quickly decreased in tissues after injection [8, 12]. *Ex vivo* EPR measurements showed that the iron oxide content of tissues quickly dropped after injection of SPIO-labeled cancer cells [8, 14]. Based on these observations, we concluded that reporter gene-based methods (e.g., bioluminescence imaging, MR reporter genes such as ferritin [29]) should be preferred to SPIO-based MR cellular imaging for the long-term tracking of metastatic cells. However, these studies did not document the fate of SPIO *in vivo*.

Here, we found that macrophages can degrade SPIO after internalization. Two complementary techniques, ICP-MS and EPR, revealed that superparamagnetic and non-superparamagnetic iron decreased in SPIO-labeled macrophages after magnetic labeling, which was associated to a 80% increase in extracellular iron 5 days after SPIO labeling. Comparatively, SPIO-labeled 4T1 breast cancer cells released low amounts of iron in the extracellular medium (17% increase in iron at day 5). The intracellular iron (oxide) content remained unchanged up to 5 days after SPIO labeling in 4T1-GFP cells. A co-culture experiment of SPIO-positive 4T1 breast cancer cells and SPIO-free J774 macrophages further revealed that macrophages engulf SPIO initially present in the cytoplasm of cancer cells. SPIO release by cancer cells is likely due to cell death and/or exocytosis of nanoparticles [30]. Our data evidence that macrophages metabolize superparamagnetic iron (MRI/EPR detectable) into non-superparamagnetic iron (MRI/EPR silent). In agreement with these results, *in vivo* MRI cell tracking experiments showed that signal voids induced by SPIO on T_2_*-w images quickly decreased after an intramuscular injection of SPIO-labeled 4T1 cells in mice. Although histology showed that remaining iron oxides were engulfed by macrophages, the use of more sensitive histological methods would perhaps highlight that some SPIO are still present in tumor cells 7 days after injection [31, 32].

We observed *in vitro* that iron oxide positive macrophages significantly degrade SPIO within 3–5 days after SPIO labeling (Figure 2). Our *in vivo* results showed that contrast loss occurred from day 2 to day 7 post-injection of SPIO positive 4T1 cancer cells. Hence, Figure 2 (iron quantification in macrophages and Figure 6 (histology) indicated that macrophages were present in the tumor and could quickly degrade SPIO. Based on these pieces of evidence, it appears that the contribution of macrophages to the *in vivo* signal loss is significant. Although histology showed that remaining iron oxides were engulfed by macrophages, the use of more sensitive histological methods would perhaps highlight that some SPIO are still present in tumor cells 7 days after injection [31, 32].

**Figure 7:**
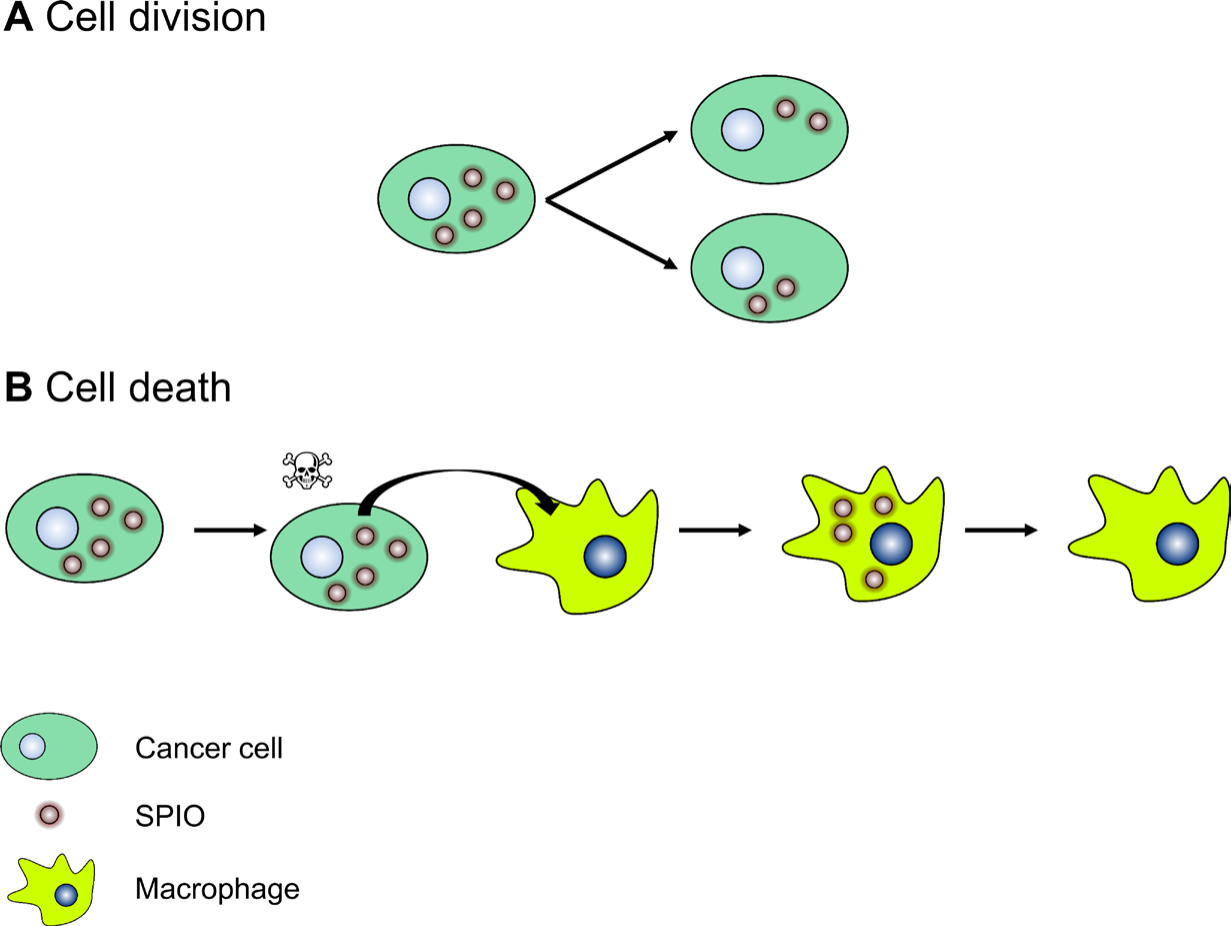
A cartoon representing the mechanisms of the loss of MR T_2_(*) contrast after injection of SPIO-labeled cancer cells *in vivo* (**A**) Dilution of the iron oxide content following cell division. For the sake of clarity, the iron oxide content is equally shared among daughter cells. (**B**) Biotransformation of SPIO to non-superparamagnetic iron by macrophages after the release of SPIO by dead cancer cells.

After *in vivo* injection of SPIO-labeled breast cancer cells, we can define two concomitant mechanisms to explain the contrast loss observed on T_2_*-w MR images (Figure 7). First, previous studies showed that the intracellular iron oxide content is shared among daughter cells during cell proliferation [10, 14, 33]. It results in a dilution of the SPIO content in the tumor and a decrease in the darkening effect of SPIO on MR T_2_-,T_2_*-w images [14, 33]. Second, the present study showed that SPIO used for cancer cell labeling is eventually taken up by macrophages. The presence of free SPIO in the tumor is likely due to cell death.

In experimental metastasis models (*i.e*., the injection of cancer cells in the blood stream), cell tracking techniques were used to ensure the proper delivery of cancer cells to targeted sites and to characterize the long-term fate of cancer cells at the metastatic site [8, 9, 12, 14, 34, 35]. In a previous study, authors evaluated the fate of breast cancer cells in the mouse brain after intracardiac injection [9]. Cells were labeled *in vitro* with nondegradable micron-sized iron oxides (MPIO) with an inert styrene/divinylbenzene coating [9]. Taking advantage of contrast changes on MR images, authors observed that the vast majority of entrapped iron-containing cancer cells died after injection (loss of negative contrast), whereas remaining cells were either dormant (signal persistence over time) or proliferating (onset of macrometastases [9]). Using a similar approach in a melanoma model, Townson *et al*. showed that treatment with doxorubicin inhibited tumor growth but did not affect the number of solitary dormant melanoma cells in the mouse liver [12]. Although our study relied on the use of degradable SPIO, we showed that tumor macrophages take up iron oxides a few days after injection of SPIO-labeled cancer cells. Hence, our works challenge the idea that MRI cellular imaging allows the proliferative status of metastatic cells to be determined.

Our results are in line with several studies that attempted to validate MR cellular imaging for tracking stem cells in cell-based therapies. Briefly, *in vivo* MRI cell tracking experiments showed that SPIO nanoparticles initially incorporated in stem cells finally end up in the cytoplasm of macrophages [36–38]. Thus, a discrepancy was reported between MRI results and the fate of the stem cells after injection *in vivo*. In our study, we observed a quick loss of the negative contrast induced by SPIO *in vivo* (< 1 week), whereas above-cited studies reported longer signal persistence after injection (4 weeks) [36]. This can be explained by differences in SPIO formulation, coating and stability.

To conclude, SPIO-based cell tracking suffers from limitations that restricts the follow-up period after injection of cells of interest *in vivo*. However, this technique is valid for ensuring the proper delivery of cells at a given site. For long-term cell tracking in preclinical models, the use of reporter genes should be preferred because their expression is not diminished after cell division and they are only expressed by living cells.

## MATERIALS AND METHODS

### Cell culture

Green fluorescent protein-expressing 4T1 murine breast cancer cells cells (4T1-GFP) were generated by lentiviral infection (addgene plasmid pRRLSIN.cPPT.PGK-GFP.WPRE #12252). 4T1-GFP cells were grown in DMEM + GlutaMAX (Invitrogen), supplemented with 10% (v/v) fetal bovine serum (Invitrogen) and 1% (v/v) penicillin/streptomycin (Invitrogen). J774 murine macrophages were grown in RPMI + GlutaMAX (Invitrogen), supplemented with 10% (v/v) fetal bovine serum (Invitrogen).

### Cell labeling with SPIO and sample preparation

On day -1, 4T1-GFP or J774 cells were seeded in 6-well plates (7.5 × 10^4^ cells/well). At day 0, the medium was removed and replaced by 2 ml of full medium containing Molday ION^™^ Rhodamine B red fluorescent SPIO particles (MIRB 25 μg Fe/ml, Biopal, Worcester, USA) for overnight incubation. MIRB are dextran-coated SPIO, have a zeta potential of ~ +31 mV and a colloidal size of 35 nm [39]. After MIRB labeling, cells were washed three times with PBS, harvested using 0.05% trypsin/EDTA (Invitrogen), spun down (180 g for 5 minutes) and resuspended in PBS. Cells were then counted using a hemocytometer, centrifuged (160 g for 3 minutes) and resuspended in PBS for EPR or ICP-MS measurements. Extracellular media were also collected at different time points after labeling and spun down for 5 minutes at 10,000 g, after which supernatants were collected for iron measurements. To characterize SPIO localization after labeling, fluorescence microscopy was performed on days 0, 1, 2, 3 and 5 post-labeling using an AxioVert S100 microscope (Zeiss). Exposure times were kept constant during experiments. All experiments were performed in triplicate.

### Iron quantification

For measuring SP iron content, samples were analyzed at room temperature using a Bruker EMX EPR spectrometer operating at 9 GHz (Bruker Biospin GmBh, Germany) with the typical parameters: 3 mT modulation amplitude, 1.724 mW power, 325.1 mT center field, and field 400 mT sweep width. When measurements are performed at room temperature, the EPR signal is specific for superparamagnetic iron [14, 20–22].

For measuring total iron (SP + non-SP) content, samples were analyzed by inductively coupled argon plasma with mass spectrometry using an Agilent 7500ce instrument as previously described [22]. Briefly, samples (50 μl) were diluted quantitatively (1:100) with a HNO_3_ 1%, HCl 0.5% solution containing all internal standards (Sc, Ge, Rh and Ir). Fe was quantified using helium mode (collision cell) and its isotope at m/z 56 with Ge (at m/z 74) as internal standard.

### Co-culture of MIRB-labeled 4T1-GFP cells and J774 macrophages and Perls' Prussian blue staining

On day -2, 4T1-GFP cells were seeded in triplicate in a 6-well plate (3 × 10^4^ cells/well). On day -1, MIRB nanoparticles were added to the culture medium (25 μg/ml, overnight incubation). On day 0, culture medium was removed, cells were washed 3 times with PBS and replenished with full medium. Fluorescence microscopy was performed to confirm the intracellular localization of MIRB particles, and 7.5 × 10^4^ control J774 macrophages were added to MIRB-labeled 4T1-GFP cells. Cells were next imaged for 2 days using fluorescence microscopy (AxioVert S100 microscope, Zeiss). Exposure times were kept constant during experiments. After fluorescence imaging, cells were rinsed 3 times with PBS, fixed with paraformaldehyde 4% (Sigma-Aldrich, Bornem, Belgium), and washed 3 times with PBS containing 0.1% Triton X-100 (Sigma-Aldrich, Bornem, Belgium) for membrane permeabilization. Fixed cells were next incubated with a PBS solution containing 1% potassium ferrocyanide (Sigma-Aldrich, Bornem, Belgium)/1% HCl for 15 minutes. After Perls' Prussian blue staining of iron, cells were washed with PBS and brightfield pictures were taken (AxioVert S100 microscope, Zeiss).

### Ethical statement

All *in vivo* experiments were performed in compliance with guidelines set by national regulations and were approved by the ethical committee for animal care of the Université catholique de Louvain (UCL).

### MRI follow-up of MIRB-labeled 4T1-GFP cells *in vivo*

Five-week-old female BALB/c mice (Janvier, Le Genest-Saint-Isle, France) were anesthetized with ketamine (80 mg/kg)/xylazine (8 mg/kg). 10^6^ MIRB-labeled 4T1-GFP cells resuspended in 100μl PBS were next injected into the right gastrocnemius muscle. 10^6^ control 4T1-GFP cells were injected into the left gastrocnemius muscle.

*In vivo* MRI experiments were performed with an 11.7 T Biospec system (Bruker, Ettlingen, Germany) with a volume transmit coil (birdcage, inner diameter = 72 mm) coupled with a receive phase array coil (with four elements) at day 0, 1, 2, 5, 6 and 7 post-injection. Animals (*n* = 4) were anesthetized by inhalation of 1.5–2% isoflurane (Forene, Abbott, Maidenhead, UK). Mouse temperature was maintained at 37.0°C using a water blanket and respiration was monitored. Coronal T_2_*-weighted (T_2_*-w) images were acquired using a multiple gradient-echo (MGE) sequence with the following parameters: TR, 1100 ms; TE, 3.36 ms; number of echoes, 16; excitation pulse angle, 30°; field of view, 3 cm × 2 cm; matrix, 386 × 256; slice thickness, 1 mm; number of slices, 15; in-plane resolution, 78 μm × 78 μm; number of averages, 3; acquisition time, 14 minutes. First-echo images were used as anatomical T_2_*-w images.

### Histological analysis of macrophages recruitment

After MRI experiments, animals were euthanized using an overdose of pentobarbital. Tumors were harvested and cut into 1 mm-thick slices, which were formalin-fixed and paraffin-embedded. Perls' Prussian blue staining and F4/80 immunostaining were performed on adjacent 5 μm sections to detect iron and macrophages, respectively. For macrophage detection, after manual deparaffinization, endogenous peroxidases were inhibited by a 20 min-treatment with 3% H_2_O_2_ in methanol. Sections were then subjected to antigen retrieval in 10 mM citrate buffer pH 5.7. Aspecific antigen binding sites were blocked with a TBS solution containing 5% BSA and 0.05% Triton. Endogenous biotin was blocked with an avidin/biotin blocking kit (Vector SP2001). Slices were stained with anti-F4/80 primary antibodies (AbDSerotec MCA497GA) overnight at 4°C at a 1/500 dilution, followed by incubation with biotinylated goat anti-rat secondary antibodies (Vector BA4001) at a 1/50 dilution for 60 min at room temperature. This reaction was visualized using Streptavidin-HRP (BD Biosciences 554066) and DAB (Dako K3468). After counterstaining with hematoxylin (Dako S3301), slices were dehydrated and coverslipped. Slides were finally digitalized at a 20× magnification with a SCN400 slide scanner (Leica, Wetzlar, Germany) and visualized on the Digital Image Hub (Leica Biosystems, Dublin, Ireland).

### Statistical analyses

All results are represented as means ± standard error of the mean (SEM). Groups were compared using One-Way ANOVA followed by Dunnett's multiple comparisons test; *p* < 0.05 was considered to be statistically significant. Statistical analyses were performed using GraphPad Prism software (GraphPad Software Inc., La Jolla, USA).

## Abbreviations

EPR: Electron paramagnetic resonance
GFP: Green fluorescent protein
ICP-MS: Inductively coupled plasma mass spectroscopy
MIRB: Molday ION™ Rhodamine B red fluorescent iron oxides
MRI: Magnetic resonance imaging
SEM: Standard error of the mean
SP: Superparamagnetic
SPIO: Superparamagnetic particles of iron oxides
T_2_^(*)^-w: T_2_^(*)^-weighted
